# Barriers to Psychological Help‐Seeking in Postpartum Women With Depression, Anxiety, or Comorbid Conditions: A Qualitative Exploration

**DOI:** 10.1111/jpm.70190

**Published:** 2026-08-03

**Authors:** Elīna Zelčāne, Kristīne Mārtinsone, Anita Pipere, Kristīne Vende, Ance Mestere, Elmārs Rancāns

**Affiliations:** ^1^ Department of Health Psychology and Paedagogy Rīga Stradiņš University Riga Latvia; ^2^ Department of Psychiatry and Narcology Rīga Stradiņš University Riga Latvia

**Keywords:** depression, generalised anxiety, help‐seeking, postpartum, qualitative study

## Abstract

**Introduction:**

Postpartum mental health research has traditionally focused on depression, though anxiety is gaining attention. Alarmingly, nearly half of the affected women do not seek help, leaving conditions frequently unrecognised and untreated.

**Aim:**

This study explores barriers to professional psychological help‐seeking among postpartum women in Latvia with postpartum depression (PPD), generalised anxiety disorder (GAD), or both.

**Method:**

Using the qualitative comparison groups design and thematic analysis, seventeen semi‐structured interviews were conducted, with four participants diagnosed with PPD, six with GAD, and seven with both PPD and GAD.

**Results:**

Overarching domains of help‐seeking barriers were: (1) Individual barriers (hesitation and internal rationalisations, lack of awareness and information about symptoms, stigmatisation and emotional barriers); (2) Family barriers (difficulties arranging childcare); and (3) Systemic and socioeconomic barriers (financial constraints, accessibility and systemic issues, lack of information). Specifically, women with PPD reported emotional unawareness and guilt; those with GAD feared burdening others. Comorbid cases emphasised logistical and access barriers.

**Implications and Recommendations:**

Help‐seeking behaviour in the postpartum period is influenced by individual, family, and structural factors. To improve access to psychological care, it is essential to foster maternal mental health literacy, normalise help‐seeking through public discourse, and ensure health systems proactively support and inform parents.

## Abstract (highlights)

**Accessible Summary:**

- What is known about the subject?
  ○ Postpartum depression and anxiety are prevalent but frequently underdiagnosed and undertreated: nearly 50% of women experiencing postpartum psychological difficulties do not seek professional help.
  ○ There is limited qualitative information on diagnosis‐related professional psychological help‐seeking barriers in Eastern European contexts for postpartum women.
- What does the paper add to existing knowledge?
  ○ Shared psychological help‐seeking barriers for women with postpartum depression, generalised anxiety disorder, and both conditions include low mental health literacy, perceived stigma, and the belief that emotional distress is a regular part of motherhood, which often leads women to prioritise their infant's needs over their own and not seek professional help.
  ○ Barriers to psychological help‐seeking among women with postpartum depression often include emotional unawareness, guilt, and self‐blame, while women with anxiety more frequently avoid seeking support due to fear of burdening others.
- What are the implications for practice?
  ○ Psychiatric and mental health nurses can collaborate with midwives, general practitioners, and other professionals to develop preparatory programmes for expectant mothers that include emotional health, self‐efficacy, stress management, and realistic expectations of motherhood alongside standard antenatal education.
  ○ Community‐based nursing outreach may benefit from incorporating home visits, local support groups, and collaboration with primary care to identify emotional difficulties early and provide accessible support for postpartum women, especially in rural areas with limited mental health services.

**Relevance Statement:**

This study examines professional psychological help‐seeking barriers among postpartum women with depression, anxiety, or both, offering insights relevant to psychiatric and mental health nursing. It emphasises the role of nurses in improving maternal mental health literacy and reducing stigma through perinatal education, particularly in collaboration with midwives, general practitioners, and psychologists. The findings highlight nurses' contributions to community outreach, especially in rural areas, as part of interdisciplinary care. Conducted in an Eastern European context, the study supports culturally responsive, nurse‐led strategies to facilitate timely, equitable access to psychological support for postpartum women.

**Key Points:**

- Help‐seeking barriers in the postpartum period are primarily shaped by psychosocial and structural factors, with diagnosis influencing how these barriers are experienced.
- Emotional unawareness and guilt were particularly prominent among women with postpartum depression, whereas fear of burdening others characterised women with generalised anxiety disorder.
- Lack of information about symptoms and available services, together with childcare and access difficulties, delayed professional psychological help‐seeking across all groups.
- Findings support routine maternal mental health discussions, early psychoeducation, and community‐based nursing initiatives to improve access to psychological care.

## 1 Introduction

The postpartum period is a psychologically sensitive phase marked by increased vulnerability to mental health difficulties. Over one‐third of women globally report health‐related complications during this time, linked to hormonal, physiological, social, and lifestyle changes (Carlson et al. 2025; WHO 2023). Postpartum depression (PPD), presenting as persistent sadness, low self‐esteem, anxiety, sleep disturbances, and difficulties bonding with the infant (NIMH 2025), and postpartum anxiety (PPA), marked by excessive worry, tension, irritability, and insomnia (Salimian et al. 2024), are among the most common disorders.

When anxiety symptoms persist for at least six months postpartum, they may indicate generalised anxiety disorder (GAD), defined by the DSM‐5 as excessive anxiety and worry across various domains (APA 2013). These symptoms may begin during pregnancy and persist into the postpartum period. This study focuses on clinically diagnosed GAD in the postpartum period, rather than treating it as a distinct postpartum‐specific disorder, thus enabling a more precise analysis of help‐seeking barriers among women experiencing clinically significant and persistent anxiety.

While PPD has traditionally dominated postpartum mental health research, increasing attention is being given to anxiety‐related symptoms, particularly in the context of the COVID‐19 pandemic and ongoing global uncertainty. Meta‐analyses report a global PPD prevalence of 26% and postpartum anxiety at 31% during the pandemic, with Europe showing particularly high rates (Caffieri et al. 2024).

Despite rising awareness, help‐seeking among postpartum women remains suboptimal due to a complex interplay of individual, familial, and systemic barriers (Place et al. 2024). Help‐seeking is defined as an adaptive coping process involving attempts to obtain external support for mental health concerns (Rickwood and Thomas 2012). Alarmingly, nearly 50% of women experiencing postpartum psychological difficulties do not seek professional help (Carlson et al. 2025; Mughal et al. 2022). This contributes to a substantial treatment gap, further exacerbated by under‐recognition of symptoms by healthcare providers (Grissette et al. 2018).

In this context, psychiatric and mental health nurses play a crucial role in postpartum care. To provide effective support, nurses must recognise key risk factors and proactively initiate sensitive conversations, as women may not openly disclose their difficulties. Psychoeducation and encouragement are particularly important in addressing stigma associated with psychological help. Therefore, identifying barriers to help‐seeking is essential for enabling nurses to deliver timely, responsive, and informed care.

Recent qualitative research (e.g., Alfayumi‐Zeadna et al. 2019; Grissette et al. 2018) highlights the complex nature of help‐seeking barriers and the importance of women's subjective and sociocultural contexts. Despite this, most studies examine PPD or anxiety separately (Hu et al. 2023; Maguire et al. 2023; Place et al. 2024), providing limited insight into barriers among women with co‐occurring conditions. Furthermore, while individual, relational, and systemic factors are linked to low help‐seeking (Grissette et al. 2018; Jones 2022; Place et al. 2024), little is known about how these barriers differ across women with PPD, GAD, or both, limiting efforts to tailor interventions. Additionally, research on professional psychological help‐seeking in Eastern Europe is scarce, with most evidence derived from Western contexts. Thus, because cultural norms and healthcare systems in post‐Soviet countries may uniquely shape help‐seeking, Latvia is a particularly relevant context for addressing this gap.

This study aims to inductively explore shared and diagnosis‐related barriers to professional psychological help‐seeking among postpartum women with PPD, GAD, or both in Latvia. To achieve this, the study is guided by two specific research questions:

What are the common barriers to seeking professional psychological help among postpartum women diagnosed with PPD, GAD, or both?

What are the diagnosis‐related barriers to seeking professional psychological help among postpartum women?

## 2 Materials and Methods

### 2.1 Methodological Perspective and Design

This qualitative study is grounded in constructivism, which served as the overarching conceptual framework. Constructivism emphasises the subjective, socially constructed nature of reality and posits that individuals interpret experiences within specific sociocultural contexts and that knowledge emerges through interaction, dialogue, and reflection (Guba and Lincoln 1994; Schwandt 2003). Within this epistemological stance, meaning is co‐constructed between the researcher and participant, with the researcher adopting a reflexive role in interpreting participants' accounts.

A qualitative research strategy was employed to explore how postpartum women make sense of emotional struggles and navigate barriers to seeking mental health care. A comparative qualitative groups design (Lindsay 2019) was used to examine the experiences of three distinct groups—women with PPD, GAD, and both conditions—enabling identification of shared and distinct patterns across cases. By comparing whole cases within their contexts, the study captures configurations that characterise each group's experiences (Palmberger and Gingrich 2014).

### 2.2 Researcher Position and Reflexivity

Acknowledging the influence of researcher positioning on interpretation (Berger and Luckmann 2016; Holmes 2020), the research team held varied standpoints. None of the researchers had personal experience with postpartum mental health difficulties, although one interviewer had previous counselling experience with postpartum women and prior experience conducting research within a constructivist paradigm, which contributed to an interpretive stance that viewed participants' accounts as subjectively constructed realities shaped by personal and sociocultural meanings rather than as objective representations of events. For example, when participants described a lack of support, the analysis distinguished between the actual absence of support and difficulties perceiving, requesting, or accepting available support. In contrast, another researcher had no prior personal or professional experience related to motherhood or depression, which may have complicated the understanding of participants' descriptions of emotional paralysis and difficulties initiating help‐seeking despite recognising distress.

Participants were not told the researchers' backgrounds, and no prior relationships existed. To minimise the risk of re‐traumatisation, interviews were carefully designed and adaptively conducted, with attention to question formulation and sequencing.

Reflexivity was maintained through post‐interview discussions and journaling to support awareness of boundaries between researcher and clinician roles. The interviewer's professional background may have influenced probing and interpretation (e.g., highlighting clinically familiar patterns), which was continuously examined through reflexive team discussions. Broad or overlapping themes were refined, divided, or merged to better capture shared patterns and differences in participants' experiences, supporting the ongoing refinement of themes throughout the analytic process. Data collection by two researchers introduced diverse perspectives on motherhood. Thematic analysis was conducted independently by two researchers and reviewed by two others, enhancing reflexivity, depth, and trustworthiness.

### 2.3 Participants and Procedure

Participants were postpartum women recruited through the broader project ‘Development of Postpartum Depression Care in Latvia’, which aimed to improve postpartum mental health care through screening validation, risk assessment, and care model development.

Women aged 18+ attending routine 4–6 week postpartum visits at Riga Maternity Hospital were screened using the Patient Health Questionnaire‐9 and Generalised Anxiety Disorder‐7. Women who scored 5 or higher on either scale were invited to undergo further assessment. Those scoring ≥ 5 were invited for further assessment, followed by remote diagnostic interviews with psychiatrists using the MINI International Neuropsychiatric Interview (M.I.N.I. 7.0.2).

During screening, participants could express interest in the qualitative component. Twenty women diagnosed with PPD, GAD, or both, and who had provided informed consent, were re‐contacted six months postpartum and invited for interviews. Inclusion criteria were: (1) having given birth in the past six months; (2) completion of initial screening and psychiatric diagnosis of PPD and/or GAD; and (3) informed consent for qualitative participation.

Of the 20 women contacted, 2 declined due to time constraints, and 1 was unreachable. The final sample comprised 17 women, aged 24–40: four with PPD, six with GAD, and seven with co‐occurring symptoms. Nine participants had their first child, five had their second, and three had their third child. Sufficient informational richness was achieved after 12 interviews, though additional interviews were conducted to improve representation, particularly of PPD. Sociodemographic data are presented in Table S1.

### 2.4 Ethical Considerations and Data Protection

The Research Ethics Committee of Rīga Stradiņš University approved this study (Nr. 2‐PĒK‐4/398/2024). Participants were informed about the study's aims, procedures, voluntary and anonymous participation, and their right to withdraw or skip distressing questions. To support emotional preparation, we provided interview questions and consent forms in advance and obtained electronic consent. Information about psychiatric support was shared. Each participant received a unique code for confidentiality, and all personal identifiers were removed from public data. Data were stored securely on the password‐protected REDCap platform and used exclusively for scientific purposes.

### 2.5 Data Collection and Procedure

Individual semi‐structured interviews were chosen as most appropriate, aligning with the qualitative comparative design and well‐suited to sensitive postpartum topics, offering flexibility beyond the interview protocol to allow follow‐up questions and foster trust and depth (Karatsareas 2022).

The interview protocol was developed and piloted by four researchers, drawing on the study's research questions, a prior scoping review on postpartum mental health (Zelčāne et al. 2026), relevant literature, and researchers' practical experience. It included questions on postpartum challenges, help‐seeking behaviours, and the evaluation of the care model. This study focuses specifically on perceived barriers to accessing professional psychological help (see File S2).

Kristīne Vende and Ance Mestere conducted interviews and led initial transcript coding. Other team members reviewed coding, helped develop the codebook, and refined themes, enhancing analytical rigour. Before data collection, all team members met to ensure a shared understanding of the guide and consistent interviews.

Participants were contacted by email or phone. Data collection took place from October 21, 2024, to May 29, 2025. Remote interviews, each lasting 40–60 min, were conducted via Zoom in Latvian or Russian by language‐matched researchers. No other individuals were present during the interviews. Sessions were video‐recorded, then transcribed and reviewed by interviewers for accuracy and context.

### 2.6 Qualitative Data Analysis

The analysis was conducted inductively, using reflexive thematic analysis (Braun and Clarke 2006). Codes and themes were developed from the data, without predefined analytic categories. In a secondary interpretive step, themes were organised into broader domains: individual, family‐related, and systemic. These domains were not predefined but constructed to capture patterns across themes and enhance conceptual clarity. As such, they provide an organising structure rather than a deductive analytic framework. This approach aligns with reflexive thematic analysis, where researchers may organise themes at different abstraction levels during later interpretation while maintaining the inductive development of themes.

Transcripts were imported into NVivo to organise coded data. The interviewers led the initial analysis, ensuring contextual sensitivity. Each researcher independently coded interviews in the language in which they were conducted, followed by a peer review. Emerging themes were refined through discussion and consensus‐building. Discrepancies were addressed through reflective dialogue.

To strengthen interpretive rigour, two additional researchers independently reviewed the final thematic structure. This analytical triangulation improved clarity, depth, and interpretive rigour.

### 2.7 Qualitative Rigour

The rigour of this study was guided by Lincoln and Guba's (1985) criteria. Credibility was supported through researcher triangulation: two researchers conducted the analysis, while two others independently reviewed the data and findings. Credibility was further enhanced by comparing transcripts across participants and including illustrative quotations. Two researchers maintained reflexive journals to document their reflections, decisions, and potential biases, in line with the Critical Appraisal Skills Programme (CASP 2025). Transferability was ensured through rich descriptions of participants' life contexts, allowing readers to assess the relevance of findings to other settings. Dependability was ensured through clear and consistent documentation of the research design, sampling, and procedures. All stages of the research process were systematically documented and justified to ensure transparency and methodological coherence. Confirmability was supported by synthesising the above criteria and maintaining ongoing reflective dialogue among team members to ensure interpretations were grounded in the data.

The study was reported in accordance with the Consolidated Criteria for Reporting Qualitative Research (COREQ). Participant checking, transcript return, and repeat interviews were not conducted due to concerns about participant burden and potential distress from revisiting sensitive postpartum experiences. Rigour and transparency were ensured through reflexive thematic analysis, including iterative coding, collaborative discussions among researchers, and systematic documentation of analytic decisions.

## 3 Results

This section addresses RQ1 (What common barriers to seeking professional psychological help are experienced by postpartum women diagnosed with PPD, GAD, or both?) and RQ2 (What diagnosis‐related barriers are experienced by each group?).

Inductive thematic analysis revealed barriers structured into three categories: individual, family‐related, and socio‐economic/systemic. For a detailed overview of themes and subthemes, see Appendix 1 ‘Thematic Structure of Findings’.

The findings are presented in two parts. First, common barriers across all diagnostic groups are examined. Next, patterns more frequently reported among women with PPD, GAD, or comorbid conditions are described. This organisation enables a comparative understanding of help‐seeking. Participant codes include a unique number and a diagnostic marker (D = depression, A = anxiety, DA = comorbid; e.g., 126DA).

### 3.1 Common Barriers Across All Diagnostic Groups

Although clinically diagnosed differently, women from all groups reported similar barriers to accessing professional psychological help.

#### 3.1.1 Individual Barriers

Thematic analysis identified three shared subthemes across women with PPD, GAD, and comorbid conditions: (1) help‐seeking hesitation and internal rationalisations, (2) lack of awareness and information about symptoms, and (3) stigmatisation and emotional barriers. Regarding help‐seeking hesitation and internal rationalisations, participants commonly referred to difficulties initiating help‐seeking: *Even just picking up the phone, going online, and googling where to find a psychologist—sometimes you are not even able to do that on a psychological level (79D). Getting over that initial hurdle—actually going—is the hardest part. You know you should do it, but somehow, getting yourself to the doctor is difficult (37A). When you are truly motivated, you go and take action. However, when I am genuinely overwhelmed, I cannot seem to feel motivated at all (126DA)*.

Additionally, interviewees acknowledged their belief in self‐reliance and the expectation that symptoms would eventually resolve without professional intervention: *Mothers are expected to be strong. They are supposed to understand that this is just a phase and that it will all eventually pass (118DA); I subconsciously tell myself that I can handle it on my own and that I will get through it. It was hard for me to accept help even from those around me (9A)*.

In addition to help‐seeking and self‐reliance, women reported that they were initially unaware of their need for assistance: *I did not seek help because, at first, I did not understand what was happening to me (16D)*. For some, psychological symptoms were not recognised as signs of a mental health condition but rather perceived as a regular part of early motherhood. This misinterpretation often led to a delay in seeking support: *It did not seem that serious. If I had known it was not normal, I definitely would have sought help (87 T)*. Several women were discouraged from seeking professional psychological help due to stigmatisation and emotional barriers, such as internalised beliefs that seeking help is a sign of weakness or failure (*At that moment I felt like there was just something wrong with me (38DA)*) or feelings of shame, guilt, or mistrust toward professionals, which made it difficult to open up about their experiences (*I would experience shame in disclosing the situation fully (94 DA)*).

#### 3.1.2 Family Related Barriers

Women also described family‐related barriers influencing their ability to seek professional psychological help. A common subtheme was sufficient support from close people, which reduced the perceived need for professional psychological help: *I didn't feel that I needed it at that time. I had a friend who also had a complicated birth and a very hard postpartum period (…). I could always message her or talk to her on WhatsApp.(…) She understood what we, as women, go through (129D)*.

Another subtheme was the difficulty of arranging childcare or the fear of leaving the baby with someone else: *The lack of childcare made it difficult to attend appointments, especially during the early postpartum period, as my husband works. I cannot leave such a small baby with any of my friends, neighbours, or anyone else (16D); I simply have no one to leave the baby with (38DA). We didn't really have any support, neither for me or my husband. While others have grandmothers, friends, or relatives, we had no one. We also didn't have a babysitter, as we had recently moved to another city and didn't have access to one*. (…) *My husband spends three hours a day getting to work, so I'm alone at home with the baby 24/7 (60DA)*. One woman noted that she did not even consider leaving their infant at such a young age (*I do not want to leave my children with anyone yet because I still have to breastfeed. I tried giving her formula, but she would not take it, so I simply could not leave her, even if someone were available (157 DA)*).

#### 3.1.3 Socioeconomic and Systemic Barriers

Three interrelated subthemes were developed within this domain: (1) financial concerns, (2) accessibility and systemic issues, and (3) lack of information. To illustrate the first subtheme, some women acknowledged that professional mental health services are often too expensive: *The financial aspect is also critical; I am aware that psychotherapy can be very expensive (61A)*. Although information about psychiatric support was provided at diagnosis, two of five women citing financial barriers were unaware that services could be accessed free of charge. In addition to financial concerns, participants also reported systemic barriers, including limited accessibility and long waiting lists for mental health specialists (*My general practitioner told me about free psychological help consultations. I called, but the waiting list was six months long (157DA)*), lack of mental health specialists outside urban areas (*I would have had to travel many kilometres with the baby, and I do not drive a car myself, so getting to Riga would have been an added stress (60DA)*) or poor cooperation with healthcare professionals (*The doctor was always focused solely on the baby and never asked about my psychological state (20A)*).

Some women were unaware of available support: *I had no idea where to look for help or what to do (157DA); I could not find any information. Probably, if I had come across that information somewhere, if someone like a midwife had mentioned it, then maybe I would have reached out for support (103A)*. *It would have been beneficial if, at the maternity hospital, they had explained that it is normal to experience specific emotional difficulties, but I did not receive any information (129D)*.

Importantly, these systemic barriers were rarely experienced in isolation but were closely linked to other domains. For example, some women noted that encouragement from healthcare professionals or clear information about the availability of free psychological services could have facilitated their decision to seek support. One participant reflected: *If I had come across that information somewhere, maybe I would have realised that I needed help (103A)*. Another added: *It would have been helpful to discuss my problems with a specialist I could trust, but no such person was available (118DA)*.

### 3.2 Patterns More Commonly Reported Among Women With PPD

Although women with GAD and comorbid conditions also reported difficulties understanding their experiences, all four women with PPD described a pronounced inability to recognise their symptoms and emotional state in the early postpartum months: *Many women like me think they do not need help. However, later, when looking back, you realise that you did need that support (16D)*. Compared to women with GAD, for whom guilt was less pronounced, all women with depression described feeling guilty. They believed it was their own failure for not coping: *I felt sad—this wasn't working, that wasn't working—and then I started blaming myself for everything (16D). I felt like I could not handle anything at all (.). All those responsibilities (.). However, you can not afford to wake up in the morning and feel bad (129D)*.

### 3.3 Patterns More Commonly Reported Among Women With GAD

Reluctance to burden others emerged as a pronounced barrier among women with GAD. This concern also appeared among those with comorbid conditions, but not women diagnosed solely with PPD. In contrast, participants with depression struggled to recognise or understand their symptoms. Women with anxiety, although they acknowledged their difficulties, hesitated to seek help out of concern for troubling others. As one said, *I did not accept help from my husband's family because I did not want to seem like a mother who could not cope (61A)*. Another reflected, *Why would I trouble anyone?… You should only seek help if things are extremely bad and you are really at the point of hurting yourself (87A)*.

### 3.4 Patterns More Commonly Reported Among Women With PPD and GAD

Among women diagnosed solely with PPD or GAD, access to psychological support was often hindered by difficulties arranging childcare or reluctance to leave their baby. In contrast, women with comorbid conditions more frequently faced logistical barriers like a lack of nearby specialist services, which were intensified by heightened emotional strain. For instance, one woman shared, *I live on the fifth floor with no elevator—going outside is a significant challenge for me. It would have been helpful to speak with someone, but it was too difficult to reach that person… When my general practitioner tells me*, ‘You know, there is a psychiatrist available in Sarkandaugava,’ *it feels like going to the moon when I am feeling unwell (126DA)*. These logistical hurdles often magnified the impact of negative experiences with providers for those with comorbid conditions, whereas among women with only PPD or GAD, challenges more often revolved around childcare and separation from the baby. One woman recalled, *When I finally saw a specialist after a long wait, I did not connect with her. I could not work with her, and that made me feel like maybe I did not need help after all (157DA)*. These findings highlight how distinct barriers—logistical versus childcare—compound emotional difficulties in accessing mental health care.

## 4 Discussion

This study explored barriers to professional psychological help‐seeking among postpartum women diagnosed with PPD, GAD, or comorbid conditions, highlighting both shared and diagnosis‐related patterns. While these barriers are widely reported in the international literature, the present findings suggest that, in the Latvian context, they are shaped by specific sociocultural and structural conditions characteristic of Eastern European and post‐Soviet settings.

These findings can be framed within a socio‐ecological model, in which help‐seeking arises from interactions among intrapersonal (e.g., symptom recognition), interpersonal (e.g., fear of burdening others), and structural (e.g., access to care) factors. From a help‐seeking process perspective (Rickwood and Thomas 2012), barriers may disrupt different stages, including problem recognition, decision to seek help, and access to services. The study extends socio‐ecological help‐seeking models by showing that structural constraints (e.g., limited service access) not only restrict behaviour but also shape intrapersonal processes, reinforcing self‐reliance and normalising distress. Thus, barriers act as interrelated mechanisms across ecological levels.

While Western literature often presents help‐seeking barriers as individual‐level phenomena (e.g., attitudes, stigma, mental health literacy) (Daehn et al. 2022; Jones 2022), the findings here indicate that in post‐Soviet contexts, these barriers are more deeply rooted in sociocultural conditions. This reflects differences in healthcare infrastructure, institutional trust, and cultural norms of self‐reliance, suggesting dominant help‐seeking models may be culturally specific (Place et al. 2024).

The analysis revealed that help‐seeking challenges are shaped more by psychosocial and structural factors than by diagnosis‐related factors, as similar themes emerged across groups. Barriers result from individual, relational, and systemic dynamics. These findings align with research on postpartum help‐seeking within broader sociocultural and healthcare contexts (Dennis and Chung‐Lee 2006; Hu et al. 2023; Manso‐Córdoba et al. 2020). For women with PPD or GAD, childcare constraints (Nguyen et al. 2022) often turn help‐seeking into a logistical issue, dependent on support networks.

By including diagnostic‐group comparisons within an Eastern European setting, this study offers a context‐sensitive contribution to a field dominated by Western perspectives. The results also support frameworks suggesting systemic support can mitigate internal hesitation. Its absence may intensify intrapersonal barriers to seeking help.

In exploring diagnosis‐related barriers to professional help‐seeking (RQ2), the findings reveal both overlapping and distinct patterns across diagnostic groups. The differences observed are better understood as variations in emphasis within a shared set of challenges. The relatively small sample size means these patterns should be interpreted cautiously as indicative rather than definitive.

Women with PPD most commonly reported difficulty recognising their emotional state, as well as feelings of guilt and self‐blame. These findings highlight the importance of enhancing maternal mental health literacy and promoting emotional insight through targeted interventions (Garapati et al. 2023; Yusefi Avrand et al. 2022). Although participants did not explicitly discuss nursing care, the findings may have implications for psychiatric and mental health nursing practice, particularly regarding early identification of emotional distress, psychoeducation, and supportive communication during the postpartum period. Previous research has highlighted the potential contribution of psychiatric and mental health nurses to maternal mental health literacy and multidisciplinary postpartum support (Jacobs and Mkhize 2021; Jones 2006; Shimange et al. 2025; Xiao et al. 2025).

In contrast, women with GAD often recognised their emotional distress but avoided seeking help due to fear of burdening others. This concern may be reinforced by societal ideals that portray mothers as self‐sufficient and emotionally resilient, which discourage open expressions of need (Cacciola and Psouni 2020; Jones 2022). Although participants did not explicitly discuss early attachment experiences, research suggests that avoidant attachment styles may inhibit help‐seeking in adulthood (Cacciola and Psouni 2020; Fonseca et al. 2018). These patterns suggest that while both groups encounter emotional barriers, the underlying mechanisms differ, necessitating diagnosis‐sensitive approaches to intervention and support.

Rather than representing distinct barrier categories, differences between diagnostic groups reflect variations in the underlying psychological mechanisms that shape help‐seeking. For women with PPD, diminished emotional awareness and heightened self‐blame suggest disruptions in self‐recognition and self‐legitimation of need. In contrast, women with GAD recognise distress but are inhibited by concern over interpersonal consequences, such as burdening others. Comorbidity suggests a cumulative burden, in which distress amplifies perceived structural barriers. This interpretation shifts the focus from diagnosis‐specific barriers to diagnosis‐related pathways that constrain help‐seeking.

A notable feature of this study is its comparative, qualitative design, which includes participants with PPD, GAD, and comorbid conditions. This approach enabled a nuanced exploration of both shared and diagnosis‐related barriers to psychological help‐seeking—an area that remains underexplored in current literature. It also addresses a significant gap by examining postpartum mental health in an Eastern European context, offering culturally and structurally informed insights. Although Latvia is sometimes classified as part of Northern Europe, its post‐Soviet legacy aligns it more closely with Eastern European mental health trends.

Traditional gender roles remain prominent in many Eastern European societies, where women are expected to assume primary responsibility for childcare and emotional labour (Evagorou et al. 2016). In Latvia, norms emphasising maternal self‐reliance and emotional resilience discourage help‐seeking and normalise postpartum distress (Lazareva et al. 2026). Participants described internalising these expectations, managing difficulties on their own, and feeling guilty when considering external support. This emphasis on self‐reliance (McNamara et al. 2018) reflects broader post‐Soviet cultural norms, in which maternal endurance and emotional containment function as socially reinforced moral expectations rather than merely individual beliefs. Stigma operates not only publicly but also as an internalised self‐evaluation, where help‐seeking is equated with maternal inadequacy. This aligns with moralised constructions of motherhood, where psychological distress is perceived as a personal failure rather than a health condition (Corrigan et al. 2014).

Structural barriers intensify these challenges, including limited funding, long waiting times, regional disparities, and weak coordination between primary and mental health care—features typical of less developed Eastern European systems (Krupchanka and Winkler 2016). In Latvia, universal maternity and postpartum care exists, but standardised PPD screening is not part of routine services (Lazareva et al. 2026).

Situating these findings within the broader cultural context offers important insights for the development of mental health policy and interventions in underrepresented regions. Barriers to postpartum help‐seeking are both universal and culturally patterned, underscoring the need for context‐sensitive approaches that address structural constraints alongside culturally embedded expectations of motherhood.

Partner support and higher mental health literacy significantly facilitate professional psychological help‐seeking for women. The prevalence of shared barriers across diagnoses underscores the benefit of transdiagnostic approaches in postpartum mental health support.

To maximise impact, healthcare professionals should adopt a proactive outreach approach, particularly in rural areas where access to services is limited and psychiatric and mental health nurses can play a central role. Strengthening collaboration among general practitioners, midwives, nurses, and psychologists may help address structural and relational barriers. In parallel, targeted training and awareness initiatives are needed to improve recognition of maternal mental health needs and ensure timely, coordinated care (Daehn et al. 2022), with specific emphasis on the competencies of psychologists, psychiatrists, and mental health nurses (Camerlynck et al. 2022).

Finally, information about available mental health services should be disseminated across diverse social groups and communication channels, including social media. Although such platforms may reinforce unrealistic expectations of motherhood and increase anxiety (Kirkpatrick and Lee 2022), they also offer opportunities to normalise emotional struggles and reduce stigma through peer support and shared experiences (Moore et al. 2020; Mulcahy et al. 2015).

### 4.1 Limitations

This study has several limitations. First, as interviews were conducted several months after childbirth, participants may have experienced recall bias, potentially reconstructing or reinterpreting emotions and decisions based on their current state. Second, self‐selection may have led to underrepresentation of women with more severe symptoms or lower care engagement. Consequently, the sample may be biased towards participants who are more reflective, proactive in seeking help, or more comfortable expressing their experiences. These biases could influence the prevalence and character of the themes identified.

The small sample size limits the interpretation of differences between diagnostic groups. These differences are better seen as tendencies within the dataset, not clearly established or generalisable patterns. Additionally, member checking was not conducted, which may limit the confirmability. However, this was mitigated through reflexive journaling and researcher triangulation.

### 4.2 Future Research Directions

Future studies could benefit from longitudinal approaches that trace help‐seeking behaviour across different postpartum stages, thereby helping identify when interventions are most effective. Including partners or family members in qualitative studies will provide richer insight into relational and contextual influences on women's emotions and help‐seeking decisions, helping inform better targeted support strategies.

## 5 Conclusions

This study reveals that barriers to professional psychological help‐seeking among postpartum women arise from a complex interplay of personal, familial, and systemic factors. These factors are more influential than diagnosis‐related symptoms. Common barriers included stigma, emotional resistance, childcare difficulties, financial and access issues, and lack of awareness. Diagnosis‐related trends emerged. Women with PPD struggled more with symptom recognition and guilt. Those with GAD feared burdening others. Women with comorbid conditions highlighted logistical challenges intensified by emotional strain. These findings underscore the need to enhance maternal mental health literacy, promote proactive provider engagement, and normalise vulnerability and help‐seeking during early motherhood through public discourse.

## Author Contributions

Conceptualisation: E.Z., K.M. Methodology: E.Z., K.M., A.P. Investigation: E.Z., K.V., A.M. Formal analysis: E.Z., K.V., A.M. Supervision: K.M., A.P., E.R. Writing – original draft: E.Z., A.M. Writing – review and editing: E.Z., K.M., A.P., K.V., A.M., E.R. All authors agree to be accountable for all aspects of the work.

## Funding

This study was funded within the framework of the European Union Recovery and Resilience Mechanism Plan and the state budget‐funded project ‘RSU Internal and RSU with LSPA External Consolidation’ (No. 5.2.1.1.i.0/2/24/I/CFLA/005), implemented within the internal research and development grant ‘Development of Postpartum Depression Care in Latvia (No. RSU‐PAG‐2024/1‐0003).

## Ethics Statement

This study was approved by the Research Ethics Committee of Rīga Stradiņš University (2‐PĒK‐4/398/2024).

## Conflicts of Interest

The authors declare no conflicts of interest.

## Supporting information


**Table S1:** Sociodemographic characteristics of interviewed participants.

**File S2:** Interview protocol.

## Data Availability

The data that support the findings of this study are available on request from the corresponding author. The data are not publicly available due to privacy or ethical restrictions.

## Appendix 1 Thematic Structure of Findings

Common barriers in seeking professional psychological help among women with PPD, GAD, or both.

**Table:**

Overarching domain	Common themes	Common sub‐subthemes	Diagnosis‐specific characteristics
Individual‐level barriers	Help‐seeking hesitation and internal rationalisations (15 of 17 interviews)	Difficulties initiating help‐seeking	
		Self‐reliance	
		Expectation that symptoms would eventually resolve	
		Reluctance to be a burden to others	Particularly pronounced in cases of anxiety
		Difficulties with time management	
	Lack of awareness and information about symptoms (8 of 17)	Unawareness of symptoms or perceiving them as normal	Particularly pronounced in cases of depression
		Insufficient information about symptoms	
	Stigmatisation and emotional barriers (8 of 17)	Prejudices against seeking psychological help	
		Feelings of shame and social stigma	
		Difficulty trusting professionals	
		Feelings of guilt about one's condition	Particularly pronounced in cases of depression
Family‐related barriers	Sufficient support from family/friends as a reason not to seek professional help (6 of 17)	Sufficient support from partner	
	Difficulties arranging childcare (5 of 17)	Lack of childcare support	Particularly pronounced in cases of comorbid conditions (depression + anxiety)
		Lack of trust in others to provide childcare	
Socioeconomic and systemic barriers	Financial constraints (5 of 17)	Financial burden, concerns about costs	
	Accessibility and systemic issues (7 of 17)	Long waiting lists for publicly funded mental health specialists	
		Lack of mental health specialists outside urban areas and logistic problems	Particularly pronounced in cases of comorbid conditions (depression + anxiety)
		Poor cooperation with healthcare professionals	
	Lack of information (5 of 17)	Insufficient information about available mental health support	
