# Supplementary material for: Barriers to Psychological Help‐Seeking in Postpartum Women With Depression, Anxiety, or Comorbid Conditions: A Qualitative Exploration
Source: J Psychiatr Ment Health Nurs. 2026 Aug 3;33(6):989–99. doi: 10.1111/jpm.70190 (PMC13748699; doi:10.1111/jpm.70190)
Supplement: Supplementary file 1 [file JPM-33-989-s001.docx]

Supplementary Table 1

**Sociodemographic Characteristics of Interviewed Participants**

| **Code*** | **Date of interview** | **Interviewer** | **Interview language**** | **Age** | **Number of births** | **Diagnosis***** | **Marital status** | **Place of residence****** |
| --- | --- | --- | --- | --- | --- | --- | --- | --- |
| 9 | 21.10.2024. | A.M | LV | 25 | 1 | GAD | Married, living with a husband | CC |
| 16 | 25.10.2024. | A.M | LV | 25 | 1 | PPD | Married, living with a husband | CC |
| 20 | 01.11.2024. | K.VK | RUS | 32 | 2 | GAD | Married, living with a husband | CC |
| 37 | 12.11.2024. | A.M | LV | 38 | 2 | GAD | Unmarried, living with a partner | CC |
| 38 | 13.11.2024. | A.M | LV | 34 | 2 | PPD and GAD | Married but living separately | OC |
| 60 | 13.12.2024. | A.M | LV | 33 | 2 | PPD and GAD | Married, living with a husband | CC |
| 61 | 08.01.2025. | K.VK | RUS | 35 | 3 | GAD | Unmarried, living with a partner | OC |
| 79 | 02.01.2025. | K.VK | RUS | 24 | 1 | PPD | Married, living with a husband | CC |
| 87 | 22.01.2025. | A.M | LV | 38 | 1 | GAD | Married, living with a husband | CC |
| 94 | 19.02.2025. | A.M | LV | 28 | 1 | PPD and GAD | Unmarried, living with a partner | RA |
| 103 | 17.02.2025. | K.VK | RUS | 30 | 1 | GAD | Unmarried, living with a partner | CC |
| 118 | 28.02.2025. | A.M | LV | 26 | 1 | PPD and GAD | Married, living with a husband | LC |
| 126 | 17.04.2025 | K.VK | LV | 37 | 3 | PPD and GAD | Married, living with a husband | CC |
| 129 | 28.03.2025 | K.VK | RUS | 40 | 3 | PPD | Married, living with a husband | CC |
| 150 | 07.04.2025 | K.VK | RUS | 38 | 1 | PPD and GAD | Never been married and never lived with a partner | CC |
| 157 | 17.04.2025 | K.VK | RUS | 25 | 2 | PPD and GAD | Married, living with a husband | RA |
| 178 | 29.05.2025 | K.VK | RUS | 28 | 1 | PPD | Married, living with a husband | CC |

*Each woman's assigned code.

**"LV" indicates interviews conducted in Latvian; "RUS" indicates interviews conducted in Russian.

***PPD - Postpartum Depression; GAD - Generalised Anxiety Disorder

**** CC – capital city (Rīga); LC – large city (Daugavpils, Liepāja, Jelgava, Ventspils, Jūrmala, Rēzekne, Jēkabpils, Valmiera); OC – other city; RA – rural area
