# Supplementary material for: Barriers to Psychological Help‐Seeking in Postpartum Women With Depression, Anxiety, or Comorbid Conditions: A Qualitative Exploration
Source: J Psychiatr Ment Health Nurs. 2026 Aug 3;33(6):989–99. doi: 10.1111/jpm.70190 (PMC13748699; doi:10.1111/jpm.70190)
Supplement: Supplementary file 2 [file JPM-33-989-s002.docx]

Supplementary file 2

**Interview protocol**

**Questions about the pregnancy, childbirth, and postpartum period**

1. How would you describe the period from pregnancy to the present moment?
   ● How would you describe your pregnancy overall?
   ● What was your childbirth experience like?
   ● How would you describe your postpartum period overall?
2. What challenges have you faced, or are currently facing, during the pregnancy, childbirth, or postpartum period, and how have they affected your well-being and daily life?
   ● Please reflect on physical, emotional, financial, and social support-related difficulties that have served as obstacles and negatively impacted your well-being or daily functioning.
   ● If you experienced emotionally difficult events during pregnancy or postpartum that had a lasting impact on your well-being, please describe them in more detail.
3. What or who has been particularly helpful for you during the pregnancy, childbirth, and postpartum period?
4. What kind of support do you wish you had received, or would like to receive in the future?
5. What has helped you - or is currently helping you - cope with challenges related to postpartum depression or anxiety? Please consider both internal and external factors that have influenced your well-being.
6. Please describe everything you have tried on your own to cope with postpartum depression or anxiety.
7. Which professionals did you turn to for psychological support? If you did not seek professional help, please describe the barriers, reasons or considerations behind that decision.

**Questions about psychiatric care during pregnancy and the postpartum period**

1. How would you describe your access to psychiatric care during pregnancy and the postpartum period?
2. If applicable, what barriers did you encounter in accessing psychiatric care during pregnancy or postpartum?
3. How would you evaluate the quality of psychiatric care you received during pregnancy and postpartum?
4. How did healthcare professionals respond to your concerns and needs?
5. How would you describe the sensitivity and respect shown by the care providers?
6. How did you feel when discussing your mental health with a healthcare provider?
7. Please describe any improvements you noticed in your mental health after receiving care.
8. If the treatment or care you received had any unwanted effects or negative outcomes, please describe them.
9. In your opinion, what aspects of psychiatric care during pregnancy and postpartum could be improved?
10. Were there services you needed but did not receive? Please describe.
11. What additional resources or information would have been helpful to you during pregnancy or postpartum, or would be helpful now?
12. Is there anything else you would like to share about your experience, suggestions, or ideas for improvement?

**Questions about the acceptability of new postpartum care model** (not analysed in this study)
